# Antimicrobial activity of alexidine, chlorhexidine and cetrimide against *Streptococcus mutans* biofilm

**DOI:** 10.1186/s12941-014-0041-5

**Published:** 2014-08-20

**Authors:** Matilde Ruiz-Linares, Carmen Maria Ferrer-Luque, Teresa Arias-Moliz, Paula de Castro, Beatriz Aguado, Pilar Baca

**Affiliations:** 1 Department of Paediatric Dentistry, School of Dentistry, University of Granada, Campus de Cartuja, Colegio Máximo s/n, Granada, Spain; 2 Department of Dental Pathology and Therapeutics, School of Dentistry, University of Granada, Campus de Cartuja, Colegio Máximo s/n, Granada, Spain; 3 Department of Microbiology, School of Dentistry, University of Granada, Granada, Spain; 4 University of Granada, Granada, Spain; 5 Department of Preventive Dentistry, School of Dentistry, University of Granada, Campus de Cartuja, Colegio Máximo s/n, Granada, Spain

**Keywords:** Alexidine, Biofilm, Cetrimide, Chlorhexidine, Streptococcus mutans

## Abstract

**Background:**

The use of antimicrobial solutions has been recommended to disinfect demineralized dentin prior to placing the filling material. The aim of this study was to evaluate the ability of several antimicrobials in controlling *Streptococcus mutans *(SM) biofilm formed in dentin.

**Methods:**

Antimicrobial activity of 0.2% and 2% chlorhexidine (CHX), 0.2% cetrimide (CTR) and 0.2%, 0.5%, 1% and 2% alexidine (ALX) was assayed on 1-week SM biofilm formed on standardized coronal dentin blocks. Results of SM biofilm antimicrobial activity by different protocols were expressed as the kill percentage of biofilm and the term “eradication” was used to denote the kill of 100% of the bacterial population. To compare the efficacies of the different protocols the Student t test was used, previously subjecting data to the Anscombe transformation.

**Results:**

All ALX concentrations tested and 0.2% CTR achieved a kill percentage higher than 99%, followed by 2% CHX with percentages above 96% (no statistically significant difference among them). Whereas 2% ALX and 0.2% CTR respectively showed eradication in 10 and 9 of the twelve specimens, 0.2% CHX did not produce eradication in any case.

**Conclusions:**

The present study shows that, when used for one minute, 2% and 1% alexidine, and 0.2% cetrimide, achieve eradication of *Streptococcus mutans* biofilm in most specimens when applied to a dentin-volumetric model.

## Background

Dental caries is a chronic and transmissible disease characterized by demineralization of the tooth due to acids produced by bacteria in biofilms formed on its surface. *Streptococcus mutans* (SM) is considered one of the most cariogenic bacteria present in human dental biofilm [1] and in dentin caries lesions [1]-[3]. Its metabolic activity is closely related to the initiation and progression of dental caries [2].

The treatment of deep caries lesions has traditionally involved removal of all the soft demineralized dentin before a filling is placed. However, operative dentistry is now moving away from complete caries removal to an ultraconservative approach, preserving tooth structure and preventing pulpal injury [3]. Clinical procedures involving incomplete caries removal are advocated based on the concept that deep carious dentin lesions comprise two distinct layers: an outer layer or infected dentin, highly contaminated and not recoverable, and an inner layer of affected dentin —less frequently contaminated with bacteria and preserving the cross-banded ultra structure of the collagen matrix— capable of being remineralized. Within this context, the objective of partial caries removal is to eliminate only superficial carious dentin that is highly infected, whereas dentin able to remineralize is maintained [4].

Microbiological and clinical studies have shown that the number of bacteria decreases during incomplete carious removal followed by adequate cavity sealing, and that lesions are clinically arrested [5]-[10]. Although a limited number of microorganisms persist under restorations a few months after the partial caries removal and sealing [5],[8],[9], some cariogenic bacteria may be found within the remaining microorganisms, such as *Streptococcus mutans*, which is currently found on sealed carious dentin [2],[6]-[9]. Moreover, it has recently been reported that the genotypic diversity of SM decreased after partial dentin removal and sealing, whereas the virulence traits of SM were unchanged, maintaining the same cariogenic potential [9].

In this context, efforts to eliminate or reduce residual bacteria in affected dentin aiming to decrease the risk of caries progression persist. Among possible strategies, antimicrobial solutions have been recommended to clean the cavity prior to introducing the filling material [10]. Of all available antimicrobials in dentistry, chlorhexidine (CHX) is still the most frequently used agent to reduce plaque aiming at caries control [11]. CHX is widely used as an antimicrobial agent for disinfection before the placement of restorations [10],[12]. It has demonstrated its effectiveness against bacteria associated with dental caries, and SM has been shown to be particularly sensitive to CHX [13]. Due to its broad antimicrobial spectrum (i.e. against gram positive/negative bacteria and fungi), CHX has been used to adjunctively treat either endodontic or periodontal diseases and to arrest/prevent caries progression [11]. At the concentrations used clinically, the biocompatibility of CHX is acceptable having a low degree of toxicity [14]. In dentin samples with bacterial biofilm, 2% CHX is effective in reducing total bacteria count, SM*, lactobacilli*[15] and *Enterococcus faecalis*[16], but it does not achieve eradication. Similarly to CHX, alexidine (ALX) is a bisbiguanide disinfectant, with faster bactericidal action [17]. ALX has been previously used as a mouthwash [18] and contact lens solution [19] and is being evaluated for possible use in endodontics [20]. Both 1% and 2% ALX concentrations have shown eradication of *E. faecalis* biofilm [21] and its substantivity in dentin has been demonstrated [22]. Furthermore, as compared with CHX, ALX shows lower toxicity when applied topically to corneal tissues *in vivo*[23]. Cetrimide (CTR) is a cationic surfactant, a quaternary ammonium derivative, which has demonstrated its effectiveness against gram-positive and gram-negative bacteria, also showing antifungal activity [24]. Generally applied as a topic antiseptic, it is not toxic at the concentrations of use [25]. It is scarcely irritanting and it reduces the surface tensión of liquids, favoring their entry into places of difficult access, such as dentin tubules [26]. These features justify its inclusión as a component of irrigating solutions used in endodontics. At the concentrations used for root canal irrigation, it has less toxicity than endodontic irrigants such as sodium hypochloride [27]. In addition to eradicating *E. faecalis* biofilms *in vitro*[28] and *ex vivo*[16], it exerts residual antimicrobial activity over time [29]. As irrigating solutions in endodontics, 0.2% CTR have shown their effectiveness [16]. It was recently shown that 0.2% cetrimide provides longer substantivity than 0.2% chlorhexidine and nearly as long as that of 2% chlorhexidine in a dentin-volumetric model [29]. There is no clear evidence to date of its effectiveness as a cavity disinfectant, but it has been effective *in vitro*, combined with polyacrylic acid, in eliminating bacteria associated with residual caries [30]. The aim of this study was therefore to evaluate the antimicrobial activity of chlorhexidine, cetrimide and alexidine against *S. mutans* biofilm, using a dentin-volumetric test.

## Methods and materials

### Bacteria strain and antimicrobial solutions

The bacteria used in this study were *S. mutans* (SM) (ATCC 25175) from the Spanish Type Culture Collection (CECT, Burgasot, Valencia). The bacteria were kept in tubes containing Brain Heart Infusion (BHI) agar (Scharlau Chemie SA, Barcelona, Spain), at 4°C for further use in the experiments. From the subculture of SM*,* a 1 McFarland standard suspension was prepared in BHI broth and subsequently diluted to obtain a suspension of approximately 6×10^7^ colony-forming units per milliliter (CFU/mL).

The solutions tested were 0.2% and 2% CHX (Guinama, Alboraya, Spain), 0.2%, 0.5%, 1% and 2% ALX (Alexidine dihydrochloride, Santa Cruz Biotechnology Inc., Heidelberg, Germany) and 0.2% CTR (Sigma-Aldrich Chemie, Steinheim, Germany).

### Preparation of dentin blocks

The protocol was approved by the Ethics Committee of the University of Granada, Spain. Twenty-seven unerupted extracted third human molars were stored in 0.1% thymol solution at 4°C. In preparing the dentin blocks, we followed the methodology described in a previous article [16]. The teeth were sectioned, and the two apical thirds of the roots were discarded, as was the occlusal coronal enamel, leaving a flat coronal dentin surface. This slice was cut into serial blocks. Four dentin blocks without enamel/tooth were then adjusted using a calibrator and polished with 150-, 220-, and 600-grit silicon carbide papers to obtain 2 × 2 × 1.2 mm (width × length × height) specimens. After sterilization, they were kept in a sterile saline solution until use shortly thereafter. The smear layer formed during preparation of the dentin blocks was eliminated by submerging them in 37% orthophosphoric acid for 15 seconds, after which they were sterilized. The specimens were randomly assigned to the different groups: 0.2% and 2% CHX; 0.2%, 0.5%, 1% and 2% ALX; 0.2% CTR; and one control group. Thus, each of the four specimens per tooth was tested in a different group. Two specimens per group, obtained from four additional molars, were studied under scanning electron microscopy (SEM). After sterilization, the specimens were incubated in BHI for 24 hours at 37°C to ensure no bacterial contamination.

### Biofilm antimicrobial activity test

The wells of 96-well microtiter plates (Nunclon Delta Surface; Nunc, Roskilde, Denmark) were inoculated with 180 μL of the initial bacterial suspension. Twelve wells were inoculated with sterile BHI for the sterility control. The sterile dentin blocks were submerged in the inoculated wells, and they were incubated on a rocking table (Swing Sw 8 10000–0015; OVAN, Badalona, Spain) for 1 week at 37°C in an anaerobic atmosphere. The BHI was refreshed daily to ensure the growth of SM on the dentin blocks, and the purity of the cultures was checked at regular intervals. Dentin specimens with SM biofilm were rinsed with 120 μL 0.9% saline solution for 2 minutes to remove bacteria that were not strongly adhered to the biofilm.

The antimicrobial activity assay was performed in the 96-well microtiter plates with 100 μL of the antimicrobial solutions per well. The saline rinsed specimens, dried with sterile paper disks, were then placed in the wells in contact with the disinfecting solutions for 1 minute. After subjecting the dentin blocks to the disinfectant protocols, sterile absorbent paper disks (IVD; Becton, Dickinson and Company, Sparks, MD) were used to eliminate any excess solution from the specimens. They were placed in Eppendorf tubes with 200 μL BHI, vortexed for 10 seconds, and then sonicated for 10 minutes to ensure biofilm recovery. Disrupted biofilm cultures were diluted serially and plated for viable cell counting.

### Scanning electron microscopy

For SEM analysis, the specimens were washed in sterile phosphate-buffered saline and then fixed with a 4% glutaraldehyde solution for 24 h. After that, biofilms were dehydrated in graded ethanol series (50, 70, 90, and 100%), dried for 24 h, and sputter coated with gold-palladium. The samples were then analyzed by SEM (Hitachi, S-510, Japan) at 25 Kv.

### Statistical analysis

Results of SM biofilm antimicrobial activity by different protocols were expressed as the kill percentage of biofilm, calculated as follows: (1 – [mean CFU_antimicrobial solution_/mean CFU_control_]) × 100). The term “eradication” was used to denote the kill of 100% of the bacterial population. To compare the efficacies of the different protocols, the Student t test was used, previously subjecting data to the Anscombe transformation.

## Results

**Figure 1:**
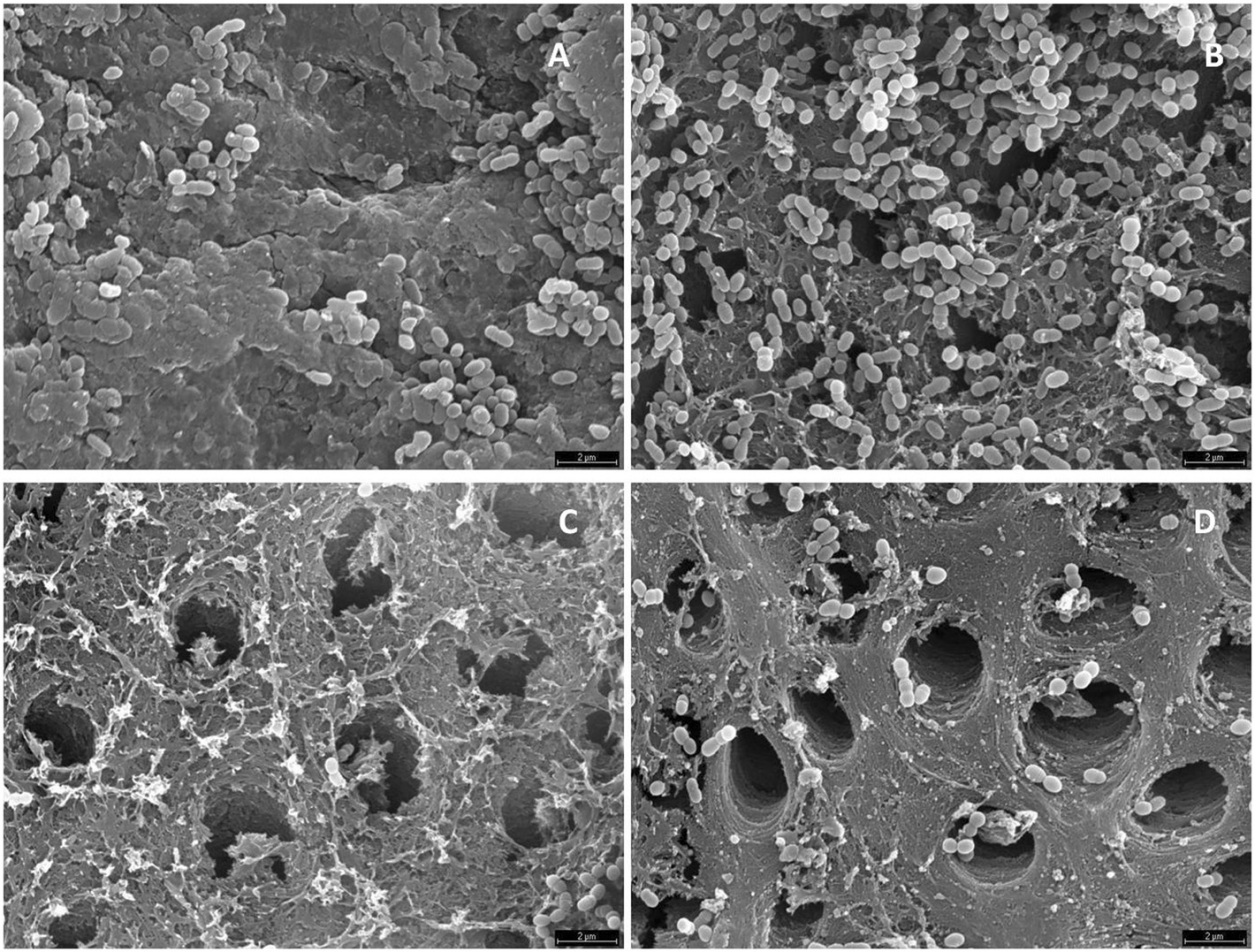
**Scanning electron microscopy images of** ***Streptococcus mutans*** **biofilm in the control group (A) and treated with 2% chlorhexidine (B), 2% Alexidine (C) and 0.2% cetrimide (D).**

The negative controls showed no bacterial growth. The results of the antimicrobial activity are shown in Table 1. All tested antimicrobial solutions obtained a high kill percentage of SM biofilms with respect to the control. Whereas 2% ALX and 0.2% CTR respectively showed eradication in 10 and 9 of the 12 specimens, 0.2% CHX did not produce eradication in any specimen. SEM images showed a reduction of bacteria within the biofilms compared with the control group (Figure 1). All the ALX concentrations tested, as well as 0.2% CTR, achieved kill percentages higher than 99%, followed by 2% CHX, with percentages above 96% (no significant statistical differences). The lowest kill percentage (85.18%), in this case proving significantly different from the other groups, was obtained with 0.2% CLX.

**Table 1:** **Antimicrobial activity against** ***Streptococcus mutans*** **biofilm of chlorhexidine, cetrimide and alexidine solutions**

**Antimicrobial solution (n = 12/group)**	**Minimum CFUs/mL**	**Maximum CFUs/mL**	**Units with E**	Kill percentage mean (standard deviation)*
2% CHX	0	61.000	6/12	96.57 (9.82)^1,3^
0.2% CHX	3.000	59.000	0/12	85.18 (10.60)^2^
2% ALX	0	19	10/12	99.99 (0.0033)^1^
1% ALX	0	72	8/12	99.99 (0.014)^1^
0.5% ALX	0	3.600	7/12	99.78 (0.58)^1,3^
0.2% ALX	0	4.700	5/12	99.56 (0.86)^3^
0.2% CTR	0	2.800	9/12	99.84 (0.45)^1,3^

CHX: chlorhexidine; ALX: alexidine; CTR: cetrimide.

E: eradication or 100% kill percentage with respect to the control.

Control values: Minimum: 60.000, maximum: 380.000, mean (standard deviation): 178.333 (105.298).

*^1, 2, 3^The same number shows differences that are not statistically significant determined by Student T test previously subjecting data to Anscombe transformation.

*p* value of comparisons: 2% CHX *vs* 0.2% CHX: *p* < 0.001, 2% CHX *vs* 2% ALX: *p* = 0.134, 2% CHX *vs* 1% ALX: *p* = 0.144, 2% CHX *vs* 0.5% ALX: *p* = 0.267, 2% CHX *vs* 0.2% ALX: *p* = 0.424, 2% CHX *vs* 0.2% CTR: *p* = 0.221, 0.2% CHX *vs* 2% ALX: p < 0.001, 0.2% CHX *vs* 1% ALX: p < 0.001, 0.2% CHX *vs* 0.5% ALX: p < 0.001, 0.2% CHX *vs* 0.2% ALX: p < 0.001, 0.2% CHX *vs* 0.2% CTR: p < 0.001, 2% ALX *vs* 1% ALX: p = 0.174, 2% ALX *vs* 0.5% ALX: p = 0.127, 2% ALX *vs* 0.2% ALX: p = 0.04, 2% ALX *vs* 0.2%CTR: p = 0.212, 1% ALX *vs* 0.5% ALX: p = 0.156, 1% ALX *vs* 0.2% ALX: p = 0.04, 1% ALX *vs* 0.2% CTR: p = 0.275, 0.5% ALX *vs* 0.2% ALX: p = 0.413, 0.5% ALX *vs* 0.2% CTR: p = 0.713, 0.2% ALX *vs* 0.2% CTR: p = 0.247.

## Discussion

Current management of deep carious lesions involves minimally invasive techniques where only a partial caries removal is performed [3]. These techniques show success in clinical studies and microbiological reports examining bacterial presence after such procedures have demonstrated that the number of microorganisms is reduced after incomplete carious dentin removal and tooth sealing [5]-[9]. However, the persistence of viable bacteria in dentin after these techniques has raised doubts regarding the long-term effectiveness of treatment [31]. *Streptococcus mutans* are cariogenic bacteria that may be found on sealed carious dentin [2],[6]-[9]. Using AP-PCR fingerprinting analysis, it has been reported that removing partially carious dentin reduces the number of genotypes of these bacteria, but not their cariogenic potential [9]. Therefore, treatment of dentin with an antibacterial agent, before sealing, is useful in eliminating the harmful effects of residual bacteria or bacterial microleakage [10]. Accordingly, our objective was to determine the ability of several antimicrobials in controlling SM biofilm formed in dentin.

Dentin represents the primary substratum for bacterial adhesion and biofilm formation [32], and its interaction with antimicrobial solutions has been clearly shown [11]. As the carrier or biological unit of biofilm formations, we chose a previously tested dentin-volumetric test [16],[22],[29] in which specimens can be easily size standardized, infected, and handled. At least four specimens may be obtained from each molar, which permits their assignment to different groups, reducing the inherent variability of the sample.

To control dentin infection, one desirable property of antimicrobial agents is their effectiveness against cariogenic bacteria growing as biofilm. In the past decade, antimicrobial agents have been incorporated into dental materials to lend them antimicrobial activity [33],[34]. The incorporation of CHX and/or CTR into glass ionomer cement (GIC) is known to confer it with beneficial antibacterial properties [34]. It was recently shown that the incorporation of CHX, CTR or both into AH Plus, an endodontic cement, improves antibacterial action against *E. faecalis*[35]. Using an Agar diffusion test, Nikihl et al. found that the addition of 2% CHX to Biodentine enhanced its antibacterial activity against SM, *S. aureus*, *E. faecalis* and *C. albicans*[36], whereas Korkmaz et al. showed that incorporating a 5%CHX-CTR mixture into conventional dental luting cement may provide greater antibacterial protection against SM and Lactobacillus [37].

For this study we selected three antimicrobial agents. CHX was included because it is considered the gold standard, functioning as a potent metalloproteinase inhibitor [38] while capable of reducing bacterial load substantially [15],[16]; yet it has not proven effective in eradicating biofilm in dentin [15], or *in vitro,* even at concentrations as high as 4% during one minute of exposure [28]. Our results confirmed the well known concentration-effectiveness of CLX and the need to use concentrations higher than the usual one (2%) to disinfect dentin. Moreover, despite the particular sensitivity of SM to CLX [16] in dentin, 0.2% CLX did not manage to eradicate the biofilm of any specimen, and the percentage of reduction (85%) was lower than with the other solutions. These results, to be expected, show that this concentration is not adequate for cavity disinfection.

The lowest kill percentages in our study were seen with CHX and the 2% CHX solution; but these did not reach statistically significant differences with respect to ALX and CTR. Six specimens showed eradication with 2% CHX, a value between those achieved with ALX at concentrations that were five (0.2% ALX) to ten times lower (0.5% ALX), indicating the great antimicrobial potential of ALX. We should stress that the form of bacteria in biofilms makes it less sensitive to antimicrobial agents, and that CHX can bind to proteins of the extracellular matrix of the biofilm, reducing its activity [39]. Moreover, dentin can inactivate or weaken the antimicrobial effect of CHX [32].

To the best of our knowledge, no studies have tested ALX as a cavity disinfectant. Nevertheless, 1% and 2% of ALX used for 1 minute provide longer antimicrobial substantivity against *E. faecalis* in dentin [22]. We found that no concentration could completely eradicate SM in all the specimens, while concentrations of 2% and 1% ALX obtained the highest kill reduction percentage (99.99%) and eradication, respectively, in 10 and 8 of the twelve specimens, giving statistically significant differences from 0.2% ALX (99.56%). Intermediate values were obtained with 0.5% ALX (99.78%). The good results with ALX in an exposure time of 1 minute might be attributed in part to the speed of its bactericidal action, given the higher affinity of ALX to lipopolysaccharide and lipoteichoic acids [40]. These results show that ALX can be an effective cavity disinfectant, particularly when techniques of partial caries removal are involved.

Cetrimide, with demonstrated effectiveness in eradicating *E. faecalis* biofilms [16] and residual antimicrobial activity [29], obtained results similar to 1% and 2% ALX. In a study using the same methodology but with *E. faecalis* biofilms, this antimicrobial solution succeeded in eradicating biofilm on all specimens [16]. The reduced effectiveness of CTR on SM could depend on the special characteristics of these bacteria. As a surfactant with antimicrobial properties which can weaken the cohesive forces of the biofilm, CTR disrupts the extracellular polysaccharide matrix [41]. The surfactant and antimicrobial properties make it effective against *E. faecalis* biofilms. Unlike *E. faecalis* however, SM can form a large quantity of extracellular polysaccharides [42], which afford this bacteria a certain ecological advantage.

We conclude that 2% and 1% ALX, as well as 0.2% CTR, achieved eradication of SM biofilm in most of the specimens of our study. In a clinical situation these antimicrobial solutions could be proposed as an alternative to CHX, for therapeutic cavity disinfection when applied for one minute. Given the diversity of the microbiota in carious dentin, and in view of the results described here, future studies should aim to evaluate the effect of different antimicrobial combinations for the control of bacteria involved in dentin caries.

## Competing interests

The authors declare no potential conflicts of interest with respect to the authorship and/or publication of this article.

## Authors’ contribution

The study was designed by authors PB, MRL and CMFL, and executed by MRL, MTAM. PC and BA participated in the study design and carried out biofilm and hard tissue preparation. The statistical analysis was done by PB and the article was written by MRL and CMFL and PB. This study is part of the doctoral thesis of BA. The acknowledged person has seen the text and given permission to be named. All authors read and approved the final manuscript.
